# Mixed-methods integration during data analysis in clinical trials of complex healthcare interventions: how is it done, and what can it do? Protocol for a systematic methodological review

**DOI:** 10.1136/bmjopen-2025-112876

**Published:** 2025-12-23

**Authors:** Holly Victoria Rose Sugg, Naomi Shaw, Ellen M Vesterlund, Joanne Woodford

**Affiliations:** 1 University of Exeter Medical School, Exeter, UK; 2 Department of Women's and Children’s Health, Uppsala University, Uppsala, Sweden; 3 School of Health Professions, University of Plymouth, Plymouth, UK

**Keywords:** Health, Clinical trials, Review, STATISTICS & RESEARCH METHODS, Feasibility Studies

## Abstract

**Abstract:**

**Introduction:**

The importance of conducting qualitative research alongside clinical trials of complex healthcare interventions is well established. There are various ways in which these two methodologies can be combined in mixed-methods research, including integrating data and/or results from the qualitative and quantitative strands during analysis, using techniques such as joint displays. The potential benefits of integration during data analysis include understanding intervention mechanisms, reasons for variation in outcomes, ways of tailoring interventions to individuals and barriers and facilitators to implementation. However, integration during data analysis may rarely be undertaken in practice, and the extent to which integration can provide valuable insights appears to be underappreciated in the field.

In this review, we aim to summarise current methods of integrating qualitative and quantitative raw data and/or results during analysis in clinical trials of complex healthcare interventions, and the yield of these different methods. Our specific research questions focus on (1) which integration techniques are used; (2) whether the results meet the study authors’ aims and/or answer their research questions; (3) the insights obtained and/or meta-inferences generated from these techniques (classified as either global or specific, and as relational, predictive, causal, comparative or elaborative); (4) any relationship between these insights and/or meta-inferences and the integration technique used and (5) the quality of these studies.

**Methods and analysis:**

We will systematically search MEDLINE, Embase, PsycINFO, CENTRAL, CINAHL, Scopus and Web of Science, and manually search reference lists. We will include studies if they integrate, during data analysis, raw data and/or results from a clinical (randomised, non-randomised or single-arm) trial and an embedded or subsequent associated qualitative study of a complex, non-pharmaceutical healthcare intervention (where the effects on a health outcome were measured). Two independent reviewers will screen titles, abstracts and full texts and perform data extraction. We will develop a descriptive account of the data, including mapping the key characteristics of included studies and narratively reporting our findings in relation to each of our research questions. We will explore how integration was undertaken, what insights were obtained and/or meta-inferences generated, and whether and how these relate to the type of integration technique used.

**Ethics and dissemination:**

This study does not require ethical approval. We intend to publish our findings in a peer-reviewed open-access journal and to present our findings at national and/or international conferences.

**Registration:**

This protocol was registered with Open Science Framework on 22 October 2025 (ref osf.io/yxtb9).

**Strengths and limitations of this study**

- To the best of our knowledge, this review is the first to focus on the value of integrating qualitative and quantitative data and/or findings specifically during data analysis.
- We include a broad and inclusive approach to searching in order to comprehensively review the whole testing, evaluation and implementation cycle of the Medical Research Council framework.
- We use the Preferred Reporting Items for Systematic review and Meta-Analysis Protocols tool, alongside the Cochrane guidance for methodological reviews, to ensure a systematic and appropriate approach to searching, screening and reporting.
- We will classify the meta-inferences generated in included studies according to published guidance in the field of mixed-methods research.
- It was not feasible to include all clinical trials of complex healthcare interventions with associated qualitative studies, as required to quantify the exact extent of missed opportunities to conduct integrative analyses in this field.

## Introduction

 The importance of combining qualitative research with clinical trials of complex healthcare interventions is ingrained within the Medical Research Council (MRC) Framework for developing and evaluating complex interventions.^1 2^ Qualitative research has a role within clinical trials conducted at the feasibility,^3 4^ evaluation^5 6^ and implementation^7^ phase, and the benefits of including qualitative research are well established.^6 8^ These include developing comprehensive and nuanced understandings of feasibility and implications for subsequent trials,^3^ explaining the mechanisms behind an intervention’s results^1^ and helping to understand trial results and the success or failure of implementation efforts.^7^

Beyond using qualitative research in clinical trials to address additional research questions and provide a comprehensive picture of an intervention in terms of both quantitative measures and participants’ subjective perspectives,^5^ mixed-methods research should ‘produce a whole… that is greater than the sum of the individual qualitative and quantitative parts’ (Fetters *et al* p116).^9^ This is achieved through integrating the qualitative and quantitative strands, which may be undertaken at the design, methodological, analysis and/or interpretation level(s), typically across sequential (explanatory or exploratory) or simultaneously conducted convergent or embedded studies.^10^

One method of integration is through data analysis. This may include integration of qualitative and quantitative results (ie, aggregated data such as themes, narrative summaries and statistics) and/or data (ie, raw data such as quotes and raw numbers).^11^ There are a variety of analytical techniques available to support this. While techniques are somewhat inconsistently referred to and categorised across the literature, examples include side-by-side comparisons, joint displays/matrices (tables or figures used to compare, synthesise or interconnect quantitative and qualitative data or results), narrative integration, data transformation and joint analyses such as following a thread, whereby initial insights from early data analysis drive the subsequent analysis of both the quantitative and qualitative data sets.^1012^,^15^

The potential benefits of integrative analyses within clinical trials of complex healthcare interventions include understanding reasons for outcome variation; exploring mechanisms of impact; identifying ways of tailoring interventions to patient preference and presentation; understanding how interventions are delivered and engaged with and exploring issues such as barriers and facilitators to implementation, the impact of context and patient experiences.^12 16^ Indeed, authors in the mixed-methods field have argued that integration during data analysis is the most important stage for maximising the explanatory power of findings from mixed-methods studies.^12 17 18^

Nonetheless, authors in the mixed-methods field suggest that, in practice, integrative analyses are rarely undertaken in clinical trials of complex healthcare interventions, with researchers typically limiting their integration of qualitative and quantitative findings to the discussion/interpretation stage, despite opportunities to do so during analysis.^16 19 20^ Any such failure to fully exploit the integrative potential of the qualitative and quantitative strands may contribute to research waste by not fully capitalising on data collected at great financial cost and participant burden.^21 22^ Thus, integrating qualitative and quantitative strands during data analysis has been highlighted as a key area for improving the conduct of mixed-methods research.^23^

Despite an increasing use of mixed-methods research in related fields,^24^ there have been mixed findings regarding the extent to which integrative analyses are undertaken. Younas *et al* found 17 examples of joint displays in nursing research during 2014–2018.^25^ In developing quality guidelines for mixed-methods research in intervention studies, Kopac and Hlebec (2020) reviewed several complex healthcare intervention studies, none of which detailed evidence of integration during analysis.^26^ Fabregues *et al* found evidence of integration in 27–30 mixed-methods intervention studies in children and adolescents with emotional and behavioural disorders, although this included examples of integration during interpretation/discussion only, and only one example of a joint display was identified.^27^ Most recently, Kletter *et al* found that although one-third of randomised controlled trials (RCTs) with nested process evaluations published since 2015 reported triangulation of process evaluation data with RCT findings, there was very limited evidence of data integration in the results sections, nor information in the methods sections as to how data triangulation took place.^28^

No reviews have focused on the complete breadth of complex healthcare interventions across the whole testing, evaluation and implementation cycle of the MRC framework. Furthermore, to the best of our knowledge, no reviews have focused on the value of integrating qualitative and quantitative strands specifically during data analysis, nor on the different insights (or meta-inferences^10^) which might be produced using the different integrative analytical techniques available. This is despite the calls made to raise both researchers’ and funders’ awareness of the value of integrative analyses in complex interventions research,^7 9 19^ and to provide researchers with both guidance and training to optimise the use of and learning from different analytical techniques.^16 19^ Thus, an up-to-date review is required to understand the current use and value of integrative analyses within clinical trials of complex healthcare interventions.

## Methods and analysis

### Aim

Our primary aim is to summarise and disseminate current methods of integrating qualitative and quantitative data or results during analysis in clinical trials of complex healthcare interventions, and the yield of these different methods. Our specific research questions are as follows:

1. In clinical trials of complex interventions that integrate quantitative and qualitative data or results during data analysis, which integration techniques are employed?
2. In what proportion of these studies are specific mixed-methods aim(s) and/or research question(s) provided, and do the results of data integration meet these aim(s) and/or answer these research question(s)?
3. What insights are obtained and/or meta-inferences generated from the integration of qualitative and quantitative data or results during analysis?
4. Do the resulting insights and meta-inferences relate to the specific integration technique used and, if so, how?
5. What is the quality of publications reporting clinical trials of complex interventions that integrate quantitative and qualitative data or results during data analysis?

### Inclusion criteria

We have formulated our inclusion criteria according to the Cochrane guidance for methodological reviews.^29^

#### Types of studies

Clinical trials (including randomised, non-randomised and single-arm designs) of complex, non-pharmaceutical interventions with the aim of improving or maintaining health (including, as per the WHO^30^ definition: diagnostic, medical, surgical, mental health, primary care, allied health, functioning support, rehabilitation, traditional medicine and public health interventions) in any setting (eg, hospitals, communities), published as journal articles.

#### Types of data

Clinical trials which quantitatively measure the effects of the intervention on one or more health outcomes, and which include a qualitative study either embedded within the trial or conducted following (and in relation to) the trial.

#### Types of method

Studies which have integrated raw data or results from the clinical trial and qualitative study during the data analysis phase, using any method of integration (eg, narrative integration; visual aids such as a joint displays).

#### Types of outcome measures

The study process measures of interest are type of integration technique used, the rationale provided for integration and the insights/meta-inferences obtained from integration.

### Exclusion criteria

We will exclude studies which are not written in English and for which the full text is not available. We will exclude protocol papers and conference abstracts as these lack the required detail on data analysis and results. In terms of outcome measures, we will exclude studies that only measure intermediary outcomes such as medication adherence, diet, self-efficacy or physical activity levels, unless they also measure the effect of the intervention on one or more health outcomes.

### Search strategy

We will conduct this review in line with the Cochrane guidance for methodological reviews.^29^ We will search the following databases from inception to March 2025, without limitation by language or publication date:

- CINAHL Ultimate (EBSCO).
- Cochrane Central Register of Controlled Trials (CENTRAL) (Wiley).
- MEDLINE, Embase, PsycINFO (Ovid).
- Scopus (Elsevier).
- Web of Science Core Collection (Clarivate Analytics) (Science Citation Index Expanded; Social Science Citation Index Expanded; Arts & Humanities Citation Index; Conference Proceedings Citation Index Science; Conference Proceedings Citation Index Social Science; Emerging Sources Citation Index).

We will check the reference lists of included studies and of review articles on mixed-methods studies or process evaluations in complex interventions research which are known to the protocol authors for additional studies.

Due to the difficulties of database searching for mixed-methods studies in terms of both study reporting and indexing across databases,^31 32^ we have designed a broad and inclusive search. An Information Specialist with expertise in searching for evidence syntheses developed draft search strategies. Search strategies include a combination of free-text terms (ie, terms in the title and abstract) and subject headings (eg, MeSH in MEDLINE) for mixed-methods research study designs and techniques for the integration of qualitative and quantitative data. We have included keywords related to qualitative research as well as mixed-methods research, to identify potentially relevant studies in the context of many authors not using the term ‘mixed-methods’, or related terms, to describe their methods.^27^ Search strategies were tested to ensure these retrieve relevant studies identified in scoping searches. A draft search strategy for Ovid MEDLINE is provided in box 1. We will adapt search strategies for other databases using appropriate syntax and controlled vocabulary.

Box 1 **Draft search strategy for Ovid MEDLINE**

1. randomised controlled trial.pt.
2. controlled clinical trial.pt.
3. randomised.ab.
4. placebo.ab.
5. clinical trials as topic.sh.
6. randomly.ab. 
7. trial.ti. 
8. exp clinical trial/
9. feasibility studies/or pilot projects/
10. ((feasibility or pilot) adj2 (trial* or study or studies)).ti,ab.
11. 1 or 2 or 3 or 4 or 5 or 6 or 7 or 8 or 9 or 10
12. exp animals/ not humans.sh.
13. 11 not 12
14. (mixed* adj1 method*).ti,ab.
15. (multiple adj1 method*).ti,ab.
16. exp Qualitative Research/
17. qualitative.ti,ab.
18. (process adj1 evaluation).ti,ab.
19. (implementation adj1 evaluation).ti,ab.
20. interview*.ti,ab. 
21. (focus adj group*).ti,ab. 
22. 14 or 15 or 16 or 17 or 18 or 19 or 20 or 21 
23. (embedd* adj5 (trial* or study or RCT or random*)).ti,ab. 
24. (integrat* adj3 (data or dataset* or finding* or analy* or result* or matrix or matrices)).ti,ab. 
25. ((merge* or merging) adj3 (data or dataset* or finding* or analy* or result*)).ti,ab. 
26. (assimilat* adj3 (data or dataset* or finding* or analy* or result*)).ti,ab. 
27. (triangulated or triangulation).ti,ab. 
28. (‘joint display’ or ‘joint displays’).ti,ab. 
29. (narrative* adj2 integrat*).ti,ab. 
30. (consolidated adj2 database*).ti,ab. 
31. (mixed* adj2 (matrix or matrices)).ti,ab. 
32. meta-matrix.ti,ab. 
33. (follow* adj2 thread*).ti,ab.
34. (spiralingspiralling or spiralling).ti,ab. 
35. ‘spiral model’.ti,ab.
36. ‘back and forth exchanges’.ti,ab. 
37. ‘back and forth exchange’.ti,ab. 
38. ‘assessment of fit’.ti,ab. 
39. (‘statistics by theme’ or ‘themes by statistics’ or ‘statistics by category’).ti,ab. 
40. (typolog* adj2 statistics).ti,ab. 
41. (‘case by case displays’ or ‘case by case display’).ti,ab. 
42. (‘case level comparison’ or ‘case level comparisons’).ti,ab. 
43. (transform* adj2 data*).ti,ab. 
44. (metainference* or meta-inference*).ti,ab. 
45. ‘combined qualitative and quantitative data’.ti,ab. 
46. ‘combined quantitative and qualitative data’.ti,ab. 
47. or/23–46. 
48. 13 and 22 and 47. 

### Screening

NS will collate the results of our searches in EndNote (Clarivate) and remove duplicates before exporting these results to Rayyan^33^ and removing any remaining duplicates. Screening will be undertaken in Rayyan.

Two reviewers will independently screen all records in a two-staged screening process of (1) title and abstract and (2) full text. At stage two, if any full texts are not available electronically, we will contact the first author to request these wherever possible (ie, if an email address or online profile can be identified for the author). We will consult a third reviewer to resolve any disagreements. To prevent any conflict of interest, where a member of the review team has authored a paper retrieved by the search, they will abstain from any discussions regarding its suitability for inclusion, and an alternative reviewer will be consulted. We will document the number of exclusions and (at the full-text screening stage) reasons for exclusions, presenting this in a Preferred Reporting Items for Systematic reviews and Meta-Analyses flow diagram.^34^

### Data extraction

We will develop a data extraction template to record data from included studies. Two reviewers will independently pilot the data extraction template on a random sample of 10% of included studies. We will compare our results and discuss discrepancies to facilitate completeness and consistency, ensuring that all relevant data are included. We will consult a third reviewer where agreement cannot be reached on the data to be included. HVRS will complete data extraction for the remaining studies.

We will extract the following information, where present, in an approach akin to a narrative review:^35^ bibliographic information (eg, country, date of publication); design (eg, RCT); aims/research questions; population; intervention (specified according to the elements of the template for intervention description and replication (TIDieR) checklist,^36^ which can be ascertained from the publication); outcome measures; methods of quantitative and qualitative data collection and analysis; and a summary of the study’s results and conclusions. We will extract, in detail, information on: (1) mixed-methods design; (2) mixed-methods aims and/or research questions, if any; (3) integration technique(s) used; (4) the insights obtained/meta-inferences generated from data integration and (5) each of the quality criteria specified in the Mixed Methods Appraisal Tool (MMAT),^37^ assessed following guidance provided in the MMAT.

### Data synthesis

We will develop a descriptive account of the data, including mapping the key characteristics of included studies (such as patient population, intervention type, study design and mixed-methods design) using basic numerical analysis such as percentages and graphical representations. We will narratively report our findings across the included studies in relation to each of our research questions. We will reference our data extraction charts to explore how integration was undertaken during data analysis and to describe the insights obtained as a result of data integration.

We will classify any meta-inferences generated according to published guidance.^24^ Thus, each meta-inference will be classified as either global or specific, depending on whether they are broad, generalised meta-inferences which are intended to be translated and/or extrapolated to populations beyond the specific study sample (‘global’) or meta-inferences which are generated only in relation to the directly studied populations and samples (‘specific’). We will further classify each meta-inference as relational (ie, identifying relationships between the original quantitative and qualitative inferences and/or variables and constructs), predictive (ie, forecasting future events, mechanisms or patterns which are formulated in such a way as to be open to future testing), causal (ie, demonstrating cause–effect relationships between the original quantitative variable(s) and qualitative construct(s)), comparative (ie, developed through triangulation to offer concordance, expansion, complementarity or discordance between quantitative and qualitative findings on a single topic), or elaborative (ie, offering new and latent insights into the studied phenomenon beyond the meaning of the original quantitative and qualitative inferences themselves).

Although dependent on our specific findings, we also aim to map the type of integration technique used to the type and extent of insights obtained/meta-inference(s) generated, using tables and/or visual aids (to be determined depending on the precise nature of our findings). We will consider the implications of our findings in the broader context of the field, drawing conclusions which place our findings in the context of current practice and making recommendations for future research. We will acknowledge the strengths and limitations of the review, ensuring transparency of conduct and processes, facilitating the identification of any potential impacts these may have on the strength or validity of our findings.

### Patient and public involvement

We have not been able to involve patients and the public in the design of this review.

### Study status

This review was started in February 2025 and is currently in the screening process. We intend to complete the review by July 2026.

## Ethics and dissemination

This study does not require ethical approval, consent to participate nor consent for publication. We intend to publish our findings in a peer-reviewed open-access journal and to present our findings at national and/or international conferences in the fields of qualitative and/or mixed-methods research, and/or complex healthcare interventions research.
