# Supplementary material for: Mixed-methods integration during data analysis in clinical trials of complex healthcare interventions: how is it done, and what can it do? Protocol for a systematic methodological review
Source: BMJ Open. 2025 Dec 23;15(12):e112876. doi: 10.1136/bmjopen-2025-112876 (PMC12730831; doi:10.1136/bmjopen-2025-112876)
Supplement: Supplementary file 1 [file bmjopen-2025-112876.reviewer_comments.pdf]

# PEER REVIEW HISTORY

BMJ Open publishes all reviews undertaken for accepted manuscripts. Reviewers are asked to complete a checklist review form and are provided with free text boxes to elaborate on their assessment. These free text comments are reproduced below.

## ARTICLE DETAILS

### Title (Provisional)

Mixed methods integration during data analysis in clinical trials of complex healthcare interventions: how is it done, and what can it do? Protocol for a systematic methodological review

### Authors

Sugg, Holly Victoria Rose; Shaw, Naomi; Vesterlund, Ellen M; Woodford, Joanne

---

## VERSION 1 - REVIEW

---

| | |
|---|---|
| **Reviewer** | **1** |
| **Name** | **Fretheim, Atle** |
| **Affiliation** | **Nasjonalt folkehelseinstitutt, Centre for Informed Health Choices** |
| **Date** | **18-Nov-2025** |
| **COI** | **None** |

---

This protocol is clear and concise, and addresses a topic of wide interest within the trialist-community: How best to integrate qualitative findings to supplement and enhance quantitative results from clinical trials.

I have only three comments: two microscopical language comments, that should be considered critically since my first language is not English, and a thrid comment that is somewhat more substantive.

1. In the abstract, I find the use of the word "authors" (last sentence in the first paragraph) a bit odd.

2. In the Introduction, I find the use of the word "However" (Last paragraph: "However, no reviews have focused on the complete breadth of complex healthcare interventions across the whole testing, evaluation, and implementation cycle of the MRC framework.") a bit odd too - I don't see what "However" points back on.

3. I worry that the authors risk excluding several relevant trials with the following exclusion criterion:

"We will exclude studies that only measure intermediary outcomes such as medication adherence, diet, selfefficacy, or physical activity levels, unless they also measure the effect of the intervention on one or more health outcomes."

The reason is that I suspect that many trials of e.g. quality improvement interventions will be relevant for this review (suich trials typically have accompanying process evaluations), but will in many cases only include "process outcomes", such as changes in prescribing of certain drugs, adherence to medication, blood pressure (usually not considered a "health outcome") etc. So, if you haven't already, I suggest that the authors consider revising this exclusion criterion. However, I would not insist on changing it, but leave it up to the authors.

---


**Reviewer** 2

**Name** Taylor, Julie

**Affiliation** University of Birmingham, Department of Applied Health Sciences, School of Health Sciences, College of Medicine and Health

**Date** 24-Nov-2025

**COI** None


---

This promises to be an exciting study with very helpful results. Some minot aspects for clarification:

You are searching from database inception, which seems a tremendously large timeframe. Would you not prefer to limit to (say) the last 10 years?

If based on Cochrane, out of interest, why Rayyan rather than Covidence?

Although you have tried to explain this, I am not convinced: How can you search specifically for integration during analysis? This isn’t typically in title or even abstract. How will you account for missing studies?

'Although it would have been beneficial to include all clinical trials of complex

healthcare interventions with associated qualitative studies, in order to quantify the

extent of missed opportunities to conduct integrative analyses in this field, it was not

feasible to include such a vast amount of literature in this review'. I am not clear what this means as you have implied you have done it?

I’d like a bit more detail: 'We will classify any meta-inferences generated, according to published guidance (24), as either global or specific, and as relational, predictive, causal, comparative, or elaborative'.

Well done on a timely piece.

## VERSION 1 - AUTHOR RESPONSE

3. Reviewer 1: In the abstract, I find the use of the word "authors" (last sentence in the first paragraph) a bit odd.
a. We have revised this sentence to remove this word.

4. Reviewer 1: In the Introduction, I find the use of the word "However" (Last paragraph: "However, no reviews have focused on the complete breadth of complex healthcare interventions across the whole testing, evaluation, and implementation cycle of the MRC framework.") a bit odd too - I don't see what "However" points back on.
a. We agree and have removed this word.

5. Reviewer 1: I worry that the authors risk excluding several relevant trials with the following exclusion criterion: "We will exclude studies that only measure intermediary outcomes such as medication adherence, diet, self-efficacy, or physical activity levels, unless they also measure the effect of the intervention on one or more health outcomes." The reason is that I suspect that many trials of e.g. quality improvement interventions will be relevant for this review (such trials typically have accompanying process evaluations), but will in many cases only include "process outcomes", such as changes in prescribing of certain drugs, adherence to medication, blood pressure (usually not considered a "health outcome") etc. So, if you haven't already, I suggest that the authors consider revising this exclusion criterion. However, I would not insist on changing it, but leave it up to the authors.
a. Thank you for this comment and we do agree that studies measuring intermediary/ process outcomes may include data integration. However, our initial searches of the literature suggest that an unwieldy volume of papers would be included were we to expand our inclusion criteria in this way; therefore, we have retained this focus on health outcomes to ensure our review is feasible.

6. Reviewer 2: You are searching from database inception, which seems a tremendously large timeframe. Would you not prefer to limit to (say) the last 10 years?
a. We do appreciate that this is a large timeframe. However, given our focus on complex interventions, we would at most want to limit our review to papers published since the MRC Framework for complex interventions was originally published in 2000, and our initial searches suggest we are unlikely to identify an unmanageable number of records from before this date anyway. We are keen to provide a comprehensive overview of integrative analyses in this field, and are therefore happy to proceed without restrictions on our timeframe if acceptable to the reviewers.

7. Reviewer 2: If based on Cochrane, out of interest, why Rayyan rather than Covidence?
a. We are both familiar with and able to use Rayyan free of charge.

8. Reviewer 2: Although you have tried to explain this, I am not convinced: How can you search specifically for integration during analysis? This isn’t typically in title or even abstract.

How will you account for missing studies?
a. We acknowledge the difficulties of relying on mixed methods terms for database searches and to try to minimise the risk of missing relevant studies we have been very broad in searching for potentially relevant terms, have tested our search strategies to ensure that no known papers in the field are missed using them, and will supplement our searches with checking reference lists of included studies and review articles on mixed methods research in complex healthcare interventions for any additional papers.
b. The alternative approach would be to search for all clinical trials of complex healthcare interventions which have an associated qualitative study. Whilst this was our original plan (see comment 9 below), we conducted initial searches on this basis which showed that the volume of relevant literature is far beyond the scope of this review. We therefore included additional terms to make the review feasible to conduct. We do acknowledge that some relevant papers may therefore be missed; however, we hope to still provide a comprehensive overview of the field.

9. Reviewer 2: 'Although it would have been beneficial to include all clinical trials of complex healthcare interventions with associated qualitative studies, in order to quantify the extent of missed opportunities to conduct integrative analyses in this field, it was not feasible to include such a vast amount of literature in this review'. I am not clear what this means as you have implied you have done it?
a. As noted regarding comment 8, we were unable to search for every clinical trial of a complex healthcare intervention with an associated qualitative study given the size of this field. If we had been able to do so, we would have been able to quantify exactly how many trials could have, but did not, integrate data. However, for the sake of feasibility we included additional search terms pertinent to mixed methods analysis/ integration, to reduce the number of hits retrieved. We can therefore provide an overview of mixed methods integration during analysis in this field, but not a broader overview of the extent of clinical trials which could have but did not include mixed methods integration. We hope to have clarified this in the associated limitation (p.3).

10. Reviewer 2: I’d like a bit more detail: 'We will classify any meta-inferences generated, according to published guidance (24), as either global or specific, and as relational, predictive, causal, comparative, or elaborative'.
a. We have provided additional information on p.8.
